# Furthering patient adherence: A position paper of the international expert forum on patient adherence based on an internet forum discussion

**DOI:** 10.1186/1472-6963-8-47

**Published:** 2008-02-27

**Authors:** Sandra van Dulmen, Emmy Sluijs, Liset van Dijk, Denise de Ridder, Rob Heerdink, Jozien Bensing, the International Expert Forum on Patient Adherence

**Affiliations:** 1 NIVEL (Netherlands Institute for Health Services Research), PO box 1568, 3500 BN Utrecht, The Netherlands; 2 Utrecht University, Department of Psychology and Health, PO box 80140, 3508 TC Utrecht, The Netherlands; 3 Utrecht University, Department of Pharmacoepidemiology & Pharmacotherapy, PO box 80082, 3508 TB Utrecht, The Netherlands

## Abstract

**Background:**

As the problem of patient non-adherence persists and a solution appears hard to be found, it continues to be important to look for new ways to further the issue. We recently conducted a meta-review of adherence intervention studies which yielded a preliminary agenda for future research, practice and theory development in patient adherence. The objective of the present project was to find out to what extent adherence experts consider this agenda relevant and feasible.

**Methods:**

The thirty-five corresponding authors of the review studies included in the meta-review were invited to join the International Expert Forum on Patient Adherence and to participate in a four-week web-based focus group discussion. The discussion was triggered by the points on the preliminary agenda presented as propositions to which forum members could react. Two researchers analysed the transcripts and selected relevant phrases.

**Results:**

Twenty adherence experts participated. Various ideas and viewpoints were raised. After the closure of the web-site, the expert forum was asked to authorize the synthesis of the discussion, to list the propositions in order of priority and to answer a few questions on the use of the web-based focus group as a tool to obtain expert opinions. Their ranking showed that the development of simple interventions is the most promising step to take in fostering patient adherence, preferably within a multidisciplinary setting of medical, pharmaceutical, social and technical science and, not in the least, by incorporating patients' perspectives.

**Conclusion:**

For enhancing adherence, the development of simple interventions originating from a multidisciplinary perspective including patients' input, appears most promising. Disclosing patients' perspectives requires open communication about patients' expectations, needs and experiences in taking medication and about what might help them to become and remain adherent.

## Background

Not taking medication as prescribed – taking either too little, or too much, for too short, or too long a period, at the wrong time or in an ineffective way – can have negative consequences for patients, healthcare and economy [1]. This non-adherence problem grows even more important as the burden of chronic diseases is growing worldwide [1]. Due to the scale of the problem it has been claimed that studies examining the effectiveness of adherence interventions should have priority over studies on new medical treatments [2]. Numerous interventions have been developed and implemented but the problem of non-adherence appears difficult to solve [3]. Given the weak theoretical underpinning of many adherence interventions, a fruitful step might be to find the most promising theoretical mainstreams.

Mindful of this aim we recently conducted a meta-review of high quality papers on patient adherence with the aim of identifying theoretical perspectives in successful adherence interventions [4]. In short, the meta-review revealed that most interventions are eclectic in nature and not strictly representative of one theoretical mainstream. In addition, there are effective adherence interventions, e.g. technical solutions, without an explicit theoretical explanation of the operating mechanisms; effective adherence interventions, e.g. incentives and reminders, which clearly originate from behavioral theories; theories which seem plausible for explaining but less powerful in improving adherence behavior; and a scarcity of comparative studies explicitly contrasting theoretical models or their components. These conclusions were summarized in six propositions reflecting a potential research agenda for future directions in research and theory development in patient adherence. The next step in furthering patient adherence was to find out how experts in the field valued the agenda, first by inquiring about each expert's current opinion of the agenda and the viewpoints being put forward by other experts, and next about the priority they would assign to the different points on the research agenda. The experts were asked to participate in a focus group discussion. In this position paper we present the synthesis of this discussion which was authorised by all participating experts and, subsequently, their priority assignments. In addition, the participants were asked to complete some questions about their experiences with the web-based discussion tool.

## Methods

### Participants

All thirtyfive corresponding authors of the review papers included in our recent meta-review on patient adherence [4] were invited to join the International Expert Forum on Patient Adherence and to give, in that capacity, their expert opinion on the future of research and theory development in patient adherence by participating in an Internet discussion. Initially, 25 review authors indicated their willingness to participate and of these, 20 actually did find time to do so.

### Internet forum discussion

The experts were approached by means of a focus group discussion triggered by the six propositions which resulted from the meta-review. Focus groups are often used to generate opinions and ideas about a specific topic [5]. Given the international character of adherence-research, Internet group discussions were held. The proposed methodology allows participants to join discussions in a rapid and convenient manner and evades logistic problems such as distance and transport. So, there are no time nor place constraints to join the focus group. In addition, in comparison with face-to-face group discussions, a web-based discussion has the advantage that participants feel free to present whatever information [6].

### Procedure

After small-scale pilot testing, a web-based focus group discussion was organized in the beginning of 2006. As a first step in the discussion, every member of the International Expert Forum received a private login-number to access the closed-circuit website. Participants were invited to login and react to the propositions offered and to contribute actively to the discussion that gradually unfolded. Firstly, the expert forum members were asked whether or not they endorsed the given proposition (Table 1) which was briefly explained (See Additional file 1) and, secondly, to clarify their opinion and react to the points made by others. In a second round, the different viewpoints and ideas were synthesized and the experts were asked to authorize the synthesis and to prioritize the propositions on importance ranging from 1 (relatively unimportant) to 5 (extremely important). The website was open for 4 weeks, 24 hours a day. The technological infrastructure of the website was developed within a study on communication and role delineation in paediatric oncology [7,8]. All participants responded using their full name.

**Table 1:** Number of experts^1 ^agreeing with each meta-review proposition and the priority scores assigned by 18 of the 20 participating experts listed from high to low

	Meta-review propositions	Agree N	Partly agree N	Disagree N	Priority score^2 ^Mean (sd)
1	Focus on simple interventions workable and feasible in (busy) clinical practice	12	5	0	2.7 (1.2)
2	Progress in adherence theories is to be expected from conjoint efforts of medical, pharmaceutical, social and technical scientists	11	5	1	3.29 (1.8)
3	Patient groups should (help to) develop adherence interventions	16	0	0	3.35 (1.5)
4	Adherence interventions should be limited solely to non-adherent patients	2	6	10	3.35 (1.9)
5	Current adherence theories are more successful in explaining than in improving adherence: theory development should focus on improving adherence	5	4	8	3.65 (1.8)
6	To improve adherence, changing the situation is more promising than changing the patient	4	12	2	4.5 (1.5)

^1 ^Not every expert reacted to every proposition

^2 ^Range 1 – 6; 1 indicating highest priority, 6 lowest priority

As the forum members were free to bring in whatever they wanted to the discussion, not every expert reacted to every proposition. The number of them who did is shown in Table 1. After the closure of the website, key arguments within each expert's reactions were selected. Two authors (ES and SvD) each read the transcripts and highligthed essential elements. Disagreements were discussed until consensus was reached about what to include in the synthesis. The forum discussion was triggered by the propositions that resulted from our meta-review [4]. For that reason, the propositions, and not the key arguments or topics raised, were used to structure the results described in this paper. As the experts may have presented a similar argument in response to different propositions, some doubling of information is inevitable. It was decided not to delete every overlapping phrase, because we thought it to be important to stay close to the experts' line of reasoning and wanted to show that some solutions, e.g. the importance of doctor-patient communication in case of reaching and maintaining adherence, were mentioned as being relevant in response to different propositions. In the last section, the overall key aspects are presented and discussed. The synthesis of the Internet forum discussion presented below includes the arguments mentioned either in favour of or against the proposition by the participating experts. Similar arguments mentioned by different experts are grouped and counted, individual remarks are marked by the expert's initials.

## Results

### Synthesis of Internet discussions

#### Simple interventions

The first proposition the forum members were asked to react to was: "Focus on simple interventions workable and feasible in (busy) clinical practice". Simple and down-to-earth interventions are badly needed, because "if the intervention is more complex than the problem, it has poor chances of offering a solution (MI)". The experts furthermore argue that the regimens patients nowadays have to follow are becoming increasingly complex, time-consuming and costly, "for example as a result of co-morbidity especially in the chronically ill (MvdW)". "The growing complexity merely points at the lack of understanding of adherence (EV)". Interventions should not only be simple for the professional but for the patient also (LT). In the experts' viewpoint, technical approaches should be used wherever possible, for example telephone reminders (MvE). These may reduce clinical load over time (JC). Nine experts think that usual care should be improved, for instance "by upskilling clinicians (JC)". The reason they give for this is that good communication and collaboration is "free" – except for the required additional time and effort – fits within daily clinical practice and should already be part of any clinicians' basic practice (MW). It is thereby considered to be important to "look at the existing trade-offs between effectiveness and costs (e.g. monetary, time, effort, etc.) both for the service provider and the patients (AG)". "In the field of mental health simple strategies, such as meeting the outpatient staff before hospital discharge, may improve adherence (CB)". Addressing adherence must be a routine part of the visit, just like taking blood pressure (LT). "We need a framework in our healthcare system to address adherence as we do with referring a patient to a specialist, or sending a patient to get blood work done. The assessment of adherence should be expected by the patient as well as the physician (LT)". The experts add to this that improvements of adherence should not only be part of the physicians' work, but should be the work of a multidisciplinary team. They state that there will always be patients who require more intensive (and expensive) guidance and support. Therefore, the experts consider it particularly important to identify suboptimal adherence in the earliest stages. Predictors of non-adherence and screening tools to identify groups at risk for non-adherence are therefore highly needed.

### Conjoint efforts

In response to the second proposition "Progress in adherence theories is to be expected from conjoint efforts of medical, pharmaceutical, social and technical scientists" fifteen out of the 20 experts argue that a multidisciplinary approach of the adherence problem is vital and definitely the way forward. "Technology plays a large role in improving adherence by designing simpler and more effective treatments with fewer behavioral demands and fewer adverse effects. In addition, human engineering and ergonomics might provide manufacturers with guidance concerning dosing, packaging and scheduling treatments (BH)". Five experts believe that technical solutions or innovations may provide universal interventions that remove barriers for all. In their opinion, new technologies can support healthcare providers to monitor (non-)adherence and provide opportunities to discuss it with the patient. However, nearly all experts also argue that technical solutions are "just one part of the puzzle (LT)". Psychosocial issues must be addressed as well because "the main hurdles to adherence remain behavioral and humanistic in nature (MI)". Many more factors are involved in adherence: healthcare providers, systems of care, treatment modalities, social environment and so on (TV). Asking about adherence in a non-threatening manner and monitoring it should be universally adopted (JC). One way to do so is by discussing possible treatment choices with the patient and the logic behind them (AY). The experts believe that it is more often the psychological variables that will ultimately determine lasting change. Four experts bring forward that technical solutions may differ between clinical areas. In mental healthcare, the emphasis is on psychotherapeutic strategies, such as the application of motivational strategies, which have shown at least some effect in improving adherence. Special attention should be given to, for example, vulnerable elderly people (MvE). In some clinical areas – for example type 2 diabetes care – biotechnical solutions are not immediately at hand. The emphasis is on the motivational and personal side of persons living with (such) health problems to support and empower them (HvD).

### Patient groups

All sixteen experts that responded to the third proposition "Patient groups should (help to) develop adherence interventions" agree unanimously that focusing on patients is crucial to improve adherence. They feel that time has come to consult individual patients or patient groups about their needs and wishes in relation to adherence. Patients or patient groups should help develop adherence interventions. One of the experts argues that, "for too long, we have tried to squeeze clinical populations into our existing models. Patients should be informing our models. This will surely lead to the most environmentally valid theories and solutions (MW)". In addition, it is important to inquire about the patients' reasons for non-adherence (TV) and the barriers to adherence (MvdW). Besides, certain interventions developed by certain patient groups may only be effective in a certain population (LT). Therefore, many different patient populations should be consulted and represented: patients from different backgrounds, age, gender and other socio-demographic characteristics (KS).

Another idea would be to let experienced patients help healthcare providers with their own 'tips and tricks' that helped them to adhere (MvdW). The experts furthermore argue that there is much we already know about what patients want. Patients who forget want simplified regimens and a reminder system, patients who get side effects want them to disappear, and patients who think the medication won' t help, don't want to take them after all (JC). Increasingly, reasons for non-adherence are being discussed, but so far adherence interventions are seldom tailored to the patients' wishes. Perhaps the best way to proceed concerns the adherence-experiences of the professionals themselves. Most of them have experienced (adherence to) self-administered treatments. Perhaps we should require the treatment developers to take the treatment (or facsimile/placebo) themselves for a period of time (BH).

### Non-adherent patients

Because of the complexity of identifying non-adherent patients, many experts disagree with the fourth proposition that "Adherence interventions should be limited solely to non-adherent patients". Although the experts do acknowledge that it is more efficient and affordable to focus on patients who need help, it is their opinion that providers cannot reliably distinguish adherent from non-adherent patients. One reason they give for this is that, due to the weak correlation between adherence and outcome, failed clinical progress is not a reliable indicator (BH). In addition, it is as yet unknown how much adherence is enough. "For certain drugs, sticking closely to the treatment regimen is very important, whereas for others the timing and frequency of dose taking may be much less crucial to achieve the desired treatment effect (KS)" Seven of the experts argue that there is not a clear distinction between adherent and non-adherent patients: it is a continuum. Four experts also mention that adherence fluctuates over time, so "adherent patients are also at risk of becoming non-adherent down the line (MI)". This indicates that one must also pay attention to the prevention of non-adherence (MvE), for instance by increasing patients' knowledge about disease and treatment (LM).

According to some experts, a distinction can be made between interventions which should be universally applied, for example technical solutions, and more individually tailored interventions. Especially for long term regimens, it remains important to put adherence permanently on the agenda of the doctor, even if the patient seems to be adherent (MvdW). Adherent patients should not be ignored (MI). Eight experts point to the importance of the doctor-patient communication in adherence. They consider communication "an essential piece of this puzzle (KH)". Respect for the patient and collaborative treatment goals are services which have no financial costs attached and should anyway be part of basic clinical practice (MW). Four experts add to this that the communication requires patients to feel comfortable discussing with their providers difficulties they may be having with a regimen, so that the regimens can be modified as needed. Therefore, the clinicians' awareness and practice of monitoring non-adherence should be improved (JC).

### Improving adherence

Eight experts disagree with the fifth proposition that "Current adherence theories are more successful in explaining than in improving adherence: theory development should focus on improving adherence". They notice that as yet there is hardly a sound theoretical basis for explaining adherence behaviour. Besides, five experts argue that adherence theories hardly explain the variance in adherence. They don't see "great strides forward in this area (DR)". More than half the group of experts agree that most current adherence interventions are not very successful in improving adherence either. They do acknowledge that there are a number of theories and constructs that have furthered our understanding of, for example, the cognitive processes underlying (non-)adherence (MW). However, the many theories have not led to efficacious standard interventions improving adherence nor necessarily translate directly into easy implementable effective clinical strategies (LM). So far, "researchers are only scratching the surface and shaking their heads at how complex adherence behavior really is (DR)". What is more, a lot of theories focus on cognitive processes but not on barriers (MvE). Six experts raise the issue that non-adherence is a complex phenomenon and that it is in general "notoriously difficult to change health behaviour (NB)". Non-adherence may have many different causes. The factors and barriers vary between patients or patient groups in different situations and there are many differences at individual level (MvE). Besides, many forms of non-adherence exist (LM). There is, for example, a significant distinction between intentional non-adherence (actively deciding not to follow prescriber's recommendations) and unintentional or non-planned non-adherence [9], which is not even considered in most theories (ED). These different forms of non-adherence require corresponding situation-specific interventions and an individually tailored approach (ED).

Seven experts state that theories are needed to understand the non-adherence phenomenon and interventions should be based on findings from theories explaining adherence. They, however, do acknowledge "the danger of re-focusing research efforts on purely intervention based theory, without in first instance fully understanding the primary problem (MW)". Ideally, there should be a two-way interaction between the development of theories and effective interventions (MvE).

With respect to the focus of future theory development, the following items were mentioned by more than three experts: Different forms of non-adherence; physician-patient communication; individual's specific adherence problems and perspectives. Three experts would encourage a more fundamental shift in focus. "Non-adherence should not be conceptualized as analogous to a pathology within the patient, and therefore needing to be 'cured' (NB)". Questions they would like to be answered include: What do patients want from healthcare and how well does professional care respond to this patients' perspective? To what extent does professional care really support and empower patients to find their way in healthcare, in self-care and in life? Finally, the experts ask for better studies and developing standardized definitions of adherence and reliable measurement instruments, together with conducting more multidisciplinary studies and well conducted qualitative studies for a better understanding of adherence. And, "the gap between behavioral science and biomedicine should be bridged (TV)".

### Change the situation

Some experts argue that it may be too early to agree to the sixth and last proposition that "To improve adherence, changing the situation is more promising than changing the patient". So far, neither (interventions) are doing very well and it is necessary to change both patient and environment (BH). This can, for instance, be done by providing equipment that makes it more easy to take medication or by strengthening patients' motivation to use their medication in a proper way, respectively. One of the experts remarks: " 'What makes it easier for patients to adhere' is a good starting point, but by itself it is unlikely to be sufficient to assure adherence. Those patients at high risk of nonadherence due to other factors, e.g. 'can't afford it', 'too many side effects', and those not inclined may need additional interventions or a change in the prescription (ED)".

The experts furthermore argue that technology has much to offer regarding the manipulation of the environment and other treatment factors. Reducing technical and environmental barriers to adherence is important, but some patients need additional measures. Psychological variables have been shown to be of at least equal importance in adherent behaviour which indicates that there is a place for behavioral and educational interventions (MW). "Technical solutions apply more universally whereas psychological factors are more individual and require individual assessment and targeting (LM)". In addition, at the population level, characteristics of the healthcare system may affect adherence (the way clinicians are trained, packaging or dosage regimen, costs). These causes need to be approached at system level (JC). At an individual level contributing causes vary between groups and individuals. Five experts argue that it is important to distinguish patterns or types of non-adherence to tailor interventions; for some patients, non-adherence is a rational choice, some may benefit from removing (situational) barriers and others need to be educated.

A number of experts address the provider. They argue that from the patients' point of view, "changing the situation may need to include changing the prescriber's behaviour (JC)". Prescribers should discuss (non-)adherence more openly with their patients and ask patients about adherence routinely (JC). "It is also important that health care providers change their own behavior to discuss this subject more open with the patient (MvdW)". Changing the situation also includes considering changing treatment guidelines, because "it may be more realistic to adjust treatment to the patient than to adjust the patient to the treatment (BB)".

### Prioritization of the propositions

Eighteen of the 20 experts that participated in the forum discussion prioritized one or more of the six propositions about the future of research and theory development in patient adherence which had formed the basis of the forum discussion (Table 1). The highest mean priority score was assigned to the effort that has to be put in exploring and developing simple interventions that can be easily implemented in everyday practice. The second highest priority was assigned to joining the knowledge of medical, pharmaceutical, social and technical sciences for enhancing adherence research and practice. Priority number three indicates that many experts believed that it is important to include patient perspectives in developing adherence interventions. Asking patients what could help them to become more adherent and tailoring interventions to these patients' wishes could be important steps to accomplish this objective. Limiting interventions to non-adherent patients only or focusing more on improving than explaining adherence scored a lower priority ranking. Finally, changing situational factors had the lowest priority for the experts. Not that environmental factors are considered irrelevant, but, according to the experts, because solely focusing on these factors engenders the risk of overlooking relevant patient-related (psychosocial) factors. An open eye to all contributing factors is warranted by most.

Overall, the priority rankings did not coincide completely with the extent to which the experts agreed to the propositions (Table 1). For instance, the proposition to which most experts disagreed, i.e. to apply adherence interventions solely to non-adherent patients, did not receive the lowest priority ranking. Likewise, the proposition related to initiating conjoint efforts was agreed upon by less experts than the proposition about including patient groups for developing interventions, yet did receive a higher priority ranking. This suggests that our way of gathering data allowed to distinguish between eliciting expert opinions about certain adherence-related themes and their viewpoints about what is needed to move adherence research, practice and theory a step further.

### Evaluation of the Internet expert forum discussion

The experts completed a few questions about the use of the Internet for obtaining insight into the state-of-the-art in patient adherence (Table 2). On the question how they experienced the use of the web-based discussion as a way to dig up international expert opinions, 15 of the 18 experts (83%) who answered this question replied that it was better than using an individual written format, one said it was better than face-to-face discussions, while two disagreed on the latter. An answer on the open question revealed that the Internet forum was considered "...an economic way of exchanging views on a subject in search of consensus". Most experts (76%) indicated that they accessed the website more than once. Twelve experts (71%) said that they reacted to other experts' comments in one or two of the six propositions, four experts (24%) did not react to others at all and one said to have reacted to other experts' comments in most propositions. Although the experts were asked to participate actively in the discussion, a real interaction may have to be stimulated somewhat more. As one of the experts remarked: "I don't think that they can replace face-to-face discussions – but it is useful for gathering information for future discussions". Someone else said: "I think it would be useful to explore ways to obtain more interaction among participants. A different structure of the discussion could improve the interaction and exchange of opinions among participants". Finally, the experts were asked how they had experienced the amount of time (4 weeks) to enter their comments on the Internet forum. Fifteen respondents (88%) answered that it was enough. Most propositions actually generated a considerable spread in answers of people either agreeing or disagreeing with a certain proposition.

**Table 2:** Evaluation questionnaire about the Internet forum discussion

For the purpose of evaluating the applied web-based method of raising expert opinions, please complete the following questions.
1. Did you participate in the Internet forum discussion?
□ No
□ Yes, I accessed the website once to enter my comments
□ Yes, I accessed the website more than once to enter my comments
2. Did you react on the comments put forward by other forum members?
□ No
□ Yes, in one or two propositions
□ Yes, in every proposition I reacted upon
3. How did you experience the use of this web-based discussion as a way to dig up international expert opinions?
□ No better than using an individual written format
□ No better than a face to face discussion
□ Better than using an individual written format
□ Better than a face to face discussion
4. How did you experience the amount of time (4 weeks) to enter your comments?
□ Too long
□ Enough
□ Too short
5. How did you value the instructions on the website?
□ Clear and understandable
□ Need some improvements
□ Difficult to understand
6. Do you have any comments that could help us in planning future web-based discussions?
...............................................................................................................
...............................................................................................................
...............................................................................................................

## Discussion

This position paper presented experts'opinions about the steps that need to be taken for furthering patient adherence. The development of simple interventions seems the most promising, preferably within a multidisciplinary setting of medical, pharmaceutical, social and technical science and, not in the least, by incorporating patients' perspectives.

Patient adherence is the outcome of a process in which many people are involved. This multifaceted character of adherence might explain why many experts agreed in joining the knowledge of medical, pharmaceutical, social and technical sciences for enhancing adherence research and practice. To quote one of the experts: "Medicine and pharmacy research and development identify new treatments; social science theories identify barriers to use the new treatments (i.e. explain non-adherence) and; technical sciences (e.g. ergonomics, human engineering) are important tools to remove such barriers and improve use of and adherence to the new treatments (AG)". Surprisingly, the potential for joint interventions at different levels, e.g. organizational, clinician and patient, is not mentioned by any of the experts, although this is a common approach to quality improvement in many healthcare settings.

The theoretical underpinning of developing simple interventions – priority number one in the eyes of the experts – is not straightforward. From a cognitive theoretical point of view, simpler regimes may simply be recalled better or, following economical cost-benefit principles, cause less discomfort. Within this line of research and practice biomedical models, behavioral theories as well as educational perspectives seem to come to the fore [4]. Yet, so far it has hardly been examined what specific elements underlying each theoretical perspective account for the most effectiveness.

The experts furthermore stress the importance of making the assessment of adherence routine business in clinical practice. This asks for the development of effective communication strategies that can be applied in discussing medication use and patient adherence in a non-threatening and open way. Identifying missed opportunities and best practices could be the first step. Another way could be to listen to what the patients themselves consider worthwhile interventions. After all, the meaning of 'simple' in relation to adherence interventions can only be deciphered by listening to the patient. Patients are the experts when trying to establish what constitutes a simple intervention, e.g. as being not too intrusive or invasive nor time-consuming or costly.

## Conclusion

For enhancing adherence, the development of simple interventions, a multidisciplinary perspective as well as the use of patients' input appear most promising. Eliciting patients' perspectives requires an open discussion of patients' expectations, needs and experiences in taking medication by the care providers involved and to pay attention to what patients might help to become and remain adherent. This is just as much a challenge for patients as it is for health professionals.

## Competing interests

The author(s) declare that they have no competing interests.

## Authors' contributions

JB conceived the study and developed its design. SvD co-ordinated the research team and drafted the manuscript together with ES. The other authors, LvD, DdR and RH, contributed substantially to the analysis and interpretation of the data and commented on the drafts of the manuscript.

## Pre-publication history

The pre-publication history for this paper can be accessed here:



## Supplementary Material

Additional file 1 The six propositions briefly explained. A brief description of the six propositions as presented to the expert panel Click here for file
