# Supplementary material for: Furthering patient adherence: A position paper of the international expert forum on patient adherence based on an internet forum discussion
Source: BMC Health Serv Res. 2008 Feb 27;8:47. doi: 10.1186/1472-6963-8-47 (PMC2278135; doi:10.1186/1472-6963-8-47)
Supplement: Additional file 1 — The six propositions briefly explained. A brief description of the six propositions as presented to the expert panel [file 1472-6963-8-47-S1.doc]

**Additional file 1 – The six propositions briefly explained**

**Proposition 1: Focus on simple interventions workable and feasible in (busy) clinical practice**

Adherence interventions are growing increasingly complex. As a consequence they become less workable in full-day (busy) clinical practice. Most interventions require extra staff, often enabled by research funding. Other solutions are needed in clinical practice.

Proposition 2: Progress in adherence theories is to be expected from conjoined efforts of medical, pharmaceutical, social and technical scientists

The importance of technical solutions in improving patient adherence points to new directions in theory development. Principles of technical sciences, for example from human engineering or ergonomics, could supplement the theories from medical and social sciences.

Proposition 3: Patient groups should (help to) develop adherence interventions

Most adherence interventions have been developed by healthcare providers. Although research has focused on patients’ reasons for non-adherence, patients have seldom been asked what they need to facilitate adherence. The time has come to consult patient groups about their needs and their wishes in relation to adherence.

### **Proposition 4: Adherence interventions should be limited solely to non-adherent patients**

Interventions should be reserved for patients who need it. Until now, most adherence interventions have involved both adherent and non-adherent patients, whereas about two thirds of the patients are spontaneously adherent. To them, interventions are a waste of time and money and perhaps affect their autonomy. Identifying non-adherent patients is crucial.

Proposition 5: Current adherence theories are more successful in explaining than in improving adherence: theory development should focus on improving adherence

Current adherence theories seem adequate to explain and understand adherence behaviour. They seem to be less adequate for establishing effective adherence interventions. Just as in medical sciences, developments in diagnostics are superior to developments in therapy. A shift in focus is needed in adherence theories.

Proposition 6: To improve adherence, changing the situation is more promising than changing the patient

The results indicate that practical and technical solutions and environmental adaptations are promising measures for improving adherence. Adherence should be considered in relation to a patient’s environment. The starting point should be: ‘what makes it easier for patients to adhere’?
